# Genome-Wide Scan and Test of Candidate Genes in the Snail *Biomphalaria glabrata* Reveal New Locus Influencing Resistance to *Schistosoma mansoni*

**DOI:** 10.1371/journal.pntd.0004077

**Published:** 2015-09-15

**Authors:** Jacob A. Tennessen, Kaitlin M. Bonner, Stephanie R. Bollmann, Joel A. Johnstun, Jan-Ying Yeh, Melanie Marine, Hannah F. Tavalire, Christopher J. Bayne, Michael S. Blouin

**Affiliations:** Department of Integrative Biology, Oregon State University, Corvallis, Oregon, United States of America; James Cook University, AUSTRALIA

## Abstract

**Background:**

New strategies to combat the global scourge of schistosomiasis may be revealed by increased understanding of the mechanisms by which the obligate snail host can resist the schistosome parasite. However, few molecular markers linked to resistance have been identified and characterized in snails.

**Methodology/Principal Findings:**

Here we test six independent genetic loci for their influence on resistance to *Schistosoma mansoni* strain PR1 in the 13-16-R1 strain of the snail *Biomphalaria glabrata*. We first identify a genomic region, *RADres*, showing the highest differentiation between susceptible and resistant inbred lines among 1611 informative restriction-site associated DNA (RAD) markers, and show that it significantly influences resistance in an independent set of 439 outbred snails. The additive effect of each *RADres* resistance allele is 2-fold, similar to that of the previously identified resistance gene *sod1*. The data fit a model in which both loci contribute independently and additively to resistance, such that the odds of infection in homozygotes for the resistance alleles at both loci (13% infected) is 16-fold lower than the odds of infection in snails without any resistance alleles (70% infected). Genome-wide linkage disequilibrium is high, with both *sod1* and *RADres* residing on haplotype blocks >2Mb, and with other markers in each block also showing significant effects on resistance; thus the causal genes within these blocks remain to be demonstrated. Other candidate loci had no effect on resistance, including the Guadeloupe Resistance Complex and three genes (*aif*, *infPhox*, and *prx1)* with immunological roles and expression patterns tied to resistance, which must therefore be trans-regulated.

**Conclusions/Significance:**

The loci *RADres* and *sod1* both have strong effects on resistance to *S*. *mansoni*. Future approaches to control schistosomiasis may benefit from further efforts to characterize and harness this natural genetic variation.

## Author Summary

Aquatic snails transmit schistosome blood flukes, causing a parasitic disease second only to malaria in its global health impact. The mechanisms by which some snails naturally resist infection are poorly understood, but if characterized could enable protocols to interfere with transmission of the disease. Here we identify a region of the snail genome that correlates with resistance to infection, and we examine its effect on immunity jointly with a previously described resistance gene. Variation at other candidate genes has no effect on parasite resistance in this laboratory population. The two resistance regions could serve as targets to block parasite transmission via snails.

## Introduction

Approximately one-sixth of the global burden of infectious disease in humans is due to parasites transmitted by invertebrate hosts [1]. A major avenue to combat these diseases lies in understanding the genetic and biochemical basis for natural variation in host resistance [2],[3]. The identification of resistance genes will facilitate genetic manipulation or marker-assisted selective breeding for resistance [4]. An understanding of host-parasite interactions may also suggest molecular therapeutic targets in the parasite. In addition, genes affecting host fitness and host-parasite co-evolution may serve as markers to predict and monitor host population responses to changes in parasite prevalence or virulence.

Although arthropod vectors have been the focus of most work on host resistance genetics, other important hosts include the aquatic snails that carry schistosomes. Measured in terms of healthy years of life lost, schistosomiasis ranks among the most consequential of infectious diseases, costing on the order of 10 million disability-adjusted life years [5]. Specifically, schistosomes infect over 200 million people [6],[7], causing a chronic disease burden that can be lifelong [8],[9]. Furthermore, the disease causes up to 200,000 deaths per year [10]. Treatment is usually based on regular dosing with a single drug, praziquantel [11], which is likely to become less effective as the parasite evolves drug resistance [12],[13]. Resistance of schistosomes to other antihelminthic therapeutics like artemisinin [14] is also possible. Vaccine development is proving elusive [15]. Thus, the development of alternative and complementary strategies, including those targeting the aquatic snails that serve as intermediate hosts, is crucial for controlling this disease.

A major barrier to developing new strategies to interrupt transmission is our limited understanding of the molecular pathways by which snails and parasites interact. Resistance to schistosome infections is highly heritable in snails, and resistance often appears to be due to one or a few major-effect loci [16–19]. However, there are still very few snail loci at which allelic variation is known to associate with resistance. Most genetic work has focused on the New World snail *Biomphalaria glabrata*. Expression levels of some *B*. *glabrata* genes are correlated with resistance [20–23], but it is unknown whether this variation is controlled by cis-regulatory elements, and therefore whether these genes are fundamentally a cause of resistance. The Guadeloupe Resistance Complex (GRC) is a snail genomic region with a strong effect on resistance to *Schistosoma mansoni* in a Caribbean population of *B*. *glabrata* [3], but its importance outside of this population remains to be shown. Alleles of the *sod1* gene, encoding superoxide dismutase, affect resistance to *S*. *mansoni* in the 13-16-R1 laboratory population of snails, possibly via the role this gene plays in the oxidative burst [24],[25], or perhaps owing to linkage with other immunity genes [26]. A few additional molecular markers are tied to resistance [18],[27], but the functional genes responsible for the phenotype are still unknown. These described loci explain only a minority of phenotypic variation in resistance, suggesting that additional resistance loci remain to be discovered. Not only will such markers represent progress toward the identification of causal resistance genes, they also can provide clues to these genes’ modes of action. That is, even before a functional gene is identified, a linked marker can reveal whether the gene acts dominantly, how it interacts epistatically with other genes, the magnitude of its effect, and which other genes are linked to it.

Strains of mixed geographical origin are ideal for identifying genetic markers with phenotypic effects, because large interpopulation differences will be reflected in the segregating allelic variation, most loci will be polymorphic with intermediate-frequency alleles, and because high linkage disequilibrium (LD) maximizes the power to find markers associated with functional polymorphisms. The *B*. *glabrata* strain 13-16-R1 has ancestry in both the Caribbean and Brazil [28–30] and thus shows high sequence diversity and high LD across its genome. Although *sod1* is linked to resistance in 13-16-R1 [24],[25], *sod1* explains only 4% of phenotypic variation in this lab population, so other genes are likely to be equally or more important. Expression levels at three additional genes (*aif*, *infPhox*, and *prx1*) are correlated with resistance in this population, but their causal roles remain to be demonstrated [23]. Most previous genetic work on this population has targeted candidate genes like these with putative immunological roles [23],[24],[31], but other genes with no prior expectation of immune function could be equally or more important. Here we perform a genome-wide scan to identify other molecular markers tied to resistance. We then test these alongside multiple candidate genes for a correlation with resistance to *S*. *mansoni* strain PR1 in the 13-16-R1 strain of *B*. *glabrata*.

## Methods

### Ethics statement

We used mice to maintain the schistosome parasites and to produce miracidia for challenge experiments. Infection is through contact with inoculated water and involves minimal discomfort. Infected rodents are euthanized with CO_2_ prior to showing clinical signs of disease and are dissected to recover parasitic eggs. Animal numbers were held to the minimum required for the research. The Oregon State University Institutional Animal Care and Use Committee, which adheres to Public Health Service Domestic Assurance for humane care and use of laboratory animals (PHS Animal Welfare Assurance Number A3229-01), approved this research as Animal Care and Use Proposal 4360.

### Inbred line RAD-Seq

From 52 inbred lines of *B*. *glabrata* 13-16-R1 (described in [23]), we examined 9 lines with low (<25%) susceptibility (“resistant”) and 10 lines with high (>85%) susceptibility (“susceptible”) (Table 1). The identities of these lines were largely, but not perfectly, concordant with the previously designated S and R lines in Larson et al. [23]. We genotyped a randomly chosen snail from each of these lines using RAD-Seq [32] with *SbfI* on the Illumina HiSeq 2000 at Oregon State University. We mapped reads to the *B*. *glabrata* reference genome version BglaB1 [33] with BWA [34] and converted genotypes to vcf format with SamTools [35]. We considered only codominant single-nucleotide polymorphisms (SNPs) (i.e. both alleles seen rather than presence/absence markers defined by null alleles). For each SNP, we counted the observations of the minor (less frequent) allele (heterozygous lines counted as 1, lines homozygous for the minor allele counted as 2; “minor allele count” = MAC), and we calculated the difference in this MAC between the resistant and susceptible lines. This framework is analogous to an F_ST_ outlier approach [3],[36], but because of the small sample sizes and high homozygosity of the inbred lines, we found MAC differences to be more straightforward to interpret than F_ST_. We included only SNPs with a MAC in the full dataset of at least 4, since lower MAC values would provide negligible statistical power to detect a meaningful difference between resistant and susceptible lines. We also excluded all SNPs with missing genotypes. For each “RAD site” (200bp region surrounding a RAD cut site, only retained for analysis if possessing a SNP), we identified the SNP showing the largest difference in MAC, and then we compared these SNPs among all RAD sites to find the RAD sites with the largest MAC differences. Scaffolds with RAD sites showing the largest differences in MAC were considered to be candidate resistance markers and examined further. Specifically, we designed PCR primers for two such candidate resistance markers (S1 Table). We Sanger sequenced one of them in representative snails from 51 of the inbred lines, and we genotyped both of them in an independent phenotype-genotype association test (described below). For all pairs of RAD sites, we calculated linkage disequilibrium (LD) as the correlation coefficient, r, and assessed the extent of high-LD regions by examining pairs with high (r > = 0.75) or perfect (r = 1) LD. LD blocks of interest were visualized with Haploview [37].

**Table 1:** Inbred lines examined with RAD-Seq.

Line	Susceptibility (%)	*RADres1* Genotype	*sod1* Genotype
R4	0	EE	BB
R31	2	EE	AA
R45	22.5	EE	CC
R68	0	EE	AA
R84	0	EE	BB
R95	4	EE	BB
R98	0	EE	AA
R113	2	EE	BB
R156	9	EE	AA
S26	100	FF	CC
S36	100	FF	AA
S46	87.5	EE	AA
S73	86.5	EE	AA
S74	100	EF	BD
S88	95	FF	BB
S90	100	FF	CC
S93	100	EE	CC
S132	98	FF	AA
S163	95	FF	CC

### Outbred snail phenotype-genotype association

We also chose eight candidate loci to test for an association with resistance phenotype in outbred snails (S1 and S2 Tables). Although other genes in *B*. *glabrata* have also been tied to immune function, this set of eight represented all genes previously found to show immune relevance in this particular 13-16-R1 lab population, as well as all known coding genes at which allelic variation correlates with *S*. *mansoni* resistance in any *B*. *glabrata* population. There are four *sod1* alleles (A, B, C, and D) segregating in this population [26], but because B is the allele correlated with resistance, for most analyses we grouped A, C, and D together as “nB” (not B). For clarity, we assigned each allele at each locus a distinct letter name without repeating letters among the loci examined in this study (Table 2). The loci *aif*, *infPhox*, and *prx1* were described in Larson et al. [23], but we designed new primers to target these loci (S1 Table). To represent the GRC [3], we used primers targeting Scaffold1_1732kb (*B*. *glabrata* reference genome BglaB1; S1 Table), here designating this locus *grc*. We designed primers to target the gene encoding biomphalysin (here designated *bmplys*), which is on the same scaffold as *sod1* (S1 Table). This locus is interesting because biomphalysin was recently shown to be a schistosome-killing protein [38], and it is located only ~500 Kb from *sod1* (*B*. *glabrata* reference genome BglaB1), close enough for substantial LD. Thus, it was of interest to ask if the association with resistance was higher for alleles at *bmplys* than at *sod1*. We also included the two scaffolds identified in the inbred line RAD-Seq described above (“*RADres1*” and “*RADres2*”). Genotypes at all loci were determined with Sanger sequencing or high resolution melt, except for *RADres2*, at which we observed PCR products of three different sizes (alleles G, H, and I), and confirmed by sequencing of homozygotes that they differ from each other by ≥ 118bp due to copy number variation. We were consequently able to genotype samples at *RADres2* by visual inspection of agarose gels after electrophoresis, without sequencing.

**Table 2:** Loci examined among 439 outbred snails.

Locus^a^	Scaffold^b^	N^c^	Alleles (% Frequency)^d^	Other loci in LD^e^	Infection Odds Impact^f^	References^g^
*sod1*	10	439	A (44), B (33), C (21), D (2)	*bmplys*	2.2**	[24]
*RADres1*	115	439	E (68), F (32)	*RADres2*	1.8**	this study
*RADres2*	332	439	G (73), H (16), I (11)	*RADres1*	1.7**	this study
*bmplys*	10	405	J (63), K (37)	*sod1*	1.9**	[38]
*aif*	1380	439	L (59), M (25), N (10), O (6)	none	none	[23]
*infPhox*	174	439	P (69), Q (23), R (6), S (1)	none	none	[23]
*prx1*	838	439	T (61), U (36), V (3)	none	none	[23]
*grc*	1	278	W (38), X (33), Y (29)	none	none	[3]

^a^Name of gene or marker

^b^Scaffold number in *B*. *glabrata* reference genome version BglaB1

^c^Number of genotyped and phenotyped outbred snails

^d^Frequency of each allele, named alphabetically

^e^Other loci showing significant linkage disequilibrium with each locus

^f^Multiplicative decrease in odds of infection conveyed by each copy of a resistance allele at each locus, significance (p < 0.01) conveyed with **

^g^First description of locus

We arbitrarily chose 456 outbred 4mm juvenile snails from the 13-16-R1 population, challenged them each with five *S*. *mansoni* miracidia (strain PR1), and classified them as infected or not, following methods described in Bonner et al [25]. Using DNA isolated from these phenotyped outbred snails, we amplified the eight candidate loci with PCR and genotyped them with Sanger sequencing, high resolution melt, and/or agarose gel electrophoresis. We tested for LD among all pairs of loci as described above, applying a Bonferroni correction of 300 (number of pairwise comparisons for 25 distinct alleles). We tested for a correlation with resistance using logistic regression (generalized linear model with a binomial response in R), as is standard for infection genetics (e.g. [39]), applying a Bonferroni correction of 25 (number of distinct alleles tested). We treated infection as a binary response variable, and modeled genotype as either additive (0, 1, or 2 alleles) or dominant (0 or 1). Thus, analyses were performed on the odds of infection, rather than the probability [40], although we also report and display probabilities for ease of interpretation. We ran simple logistic regression for all alleles at all eight loci (“single-allele tests”), and we ran multiple logistic regression with two independent variables for all pairs of alleles (“two-allele tests”, either for alleles at the same locus or across loci). We estimated the impact on odds of infection for each allele as the antilog of the β coefficient. We measured the proportion of variation explained (R^2^) as (Null deviance-Residual deviance)/(Null deviance). In order to test for a dominance effect, we calculated Clopper-Pearson binomial 95% confidence intervals [41] for the proportion of infected snails of each genotype, and tested whether estimates for heterozygotes and homozygotes overlapped. To test for partial dominance, we calculated the expected heterozygous effect for each locus, under additivity, as the mean between the two homozygous genotypes, based on log of odds, and tested whether this fell within the 95% confidence interval for heterozygotes. We tested for epistatic interaction by adding an interaction term to multiple logistic regression models. We further explored epistatic interaction by testing the effect of an allele at one locus in subsets of individuals with fixed genotypes at another, putatively interacting, locus.

### Accession numbers

Illumina data have been deposited at NCBI SRA, Bioproject Accession PRJNA270097.

## Results

### New candidate resistance region from inbred lines

**Fig 1:**
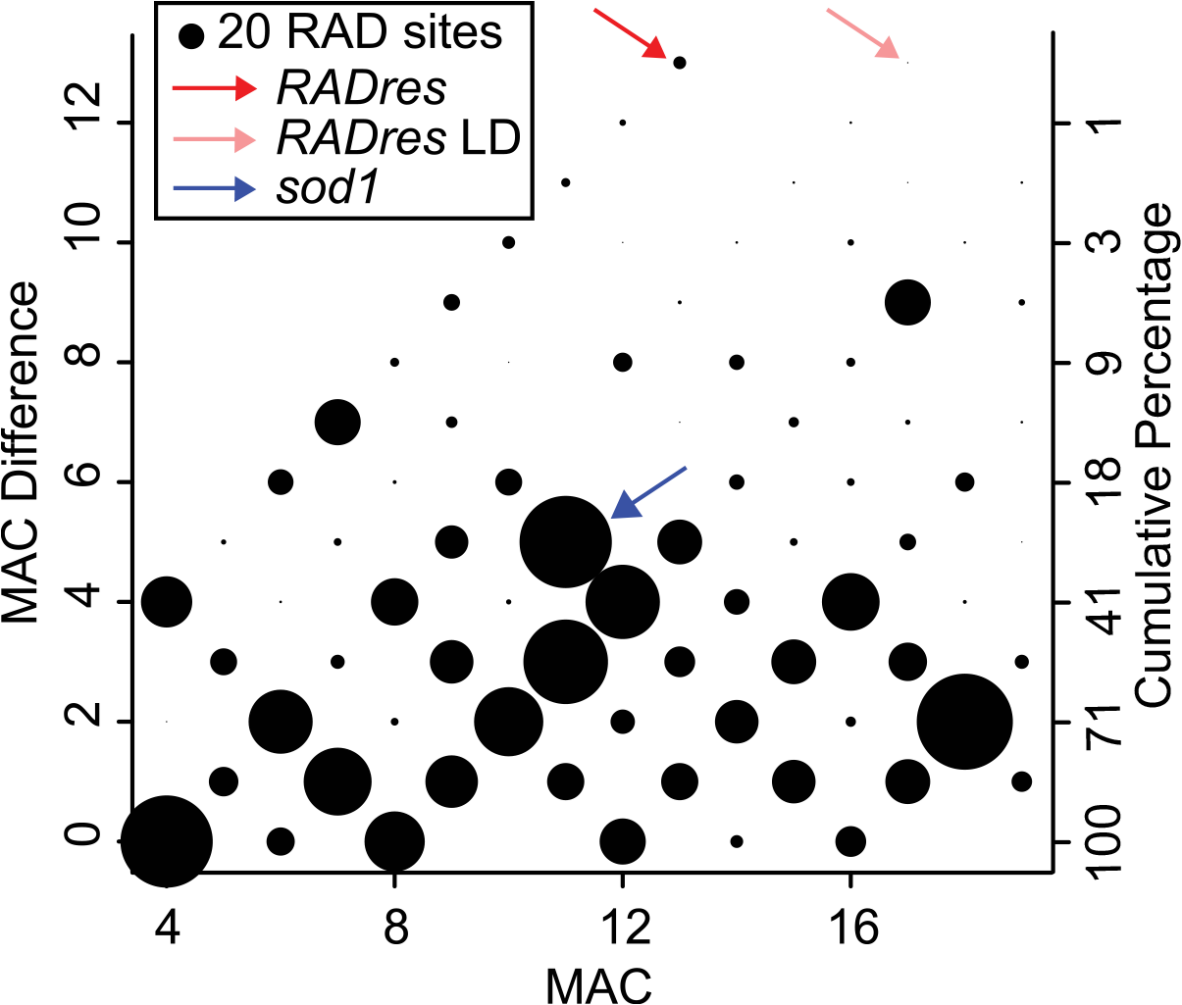
Distribution of allele frequencies among RAD sites. We characterized 1611 informative RAD sites in 19 inbred lines by minor allele count (“MAC”) (maximum of 19; i.e. 50% allele frequency) and difference in MAC between susceptible (N = 10) and resistant (N = 9) lines (“MAC difference”, theoretical maximum = 18; i.e. 9 resistant lines fixed for one allele, 10 susceptible lines fixed for another). Circle sizes are proportional to the number of RAD sites showing each pattern. The cumulative percentage of RAD sites, starting with the highest observed MAC difference, is shown on the righthand y-axis. The highest MAC difference was observed for 10 RAD sites in perfect mutual LD, with a MAC of 13 and a MAC difference of 13, which we defined as the *RADres* region and examined further (red arrow; encompasses scaffolds of subsequently examined markers *RADres1* and *RADres2*). The one remaining RAD site with an equivalent MAC difference was also in high, but not perfect, LD with *RADres* (pink arrow; scaffold not examined further). The *sod1* haplotype block had a MAC difference of 5, which was higher than average but not an outlier (blue arrow).

We observed 4304 RAD-Seq SNPs, of which 692 had a MAC under 4 and were discarded, leaving 3612 SNPs on 1611 informative RAD sites on 1259 scaffolds totaling 209Mb. These represented fewer than 1% of the scaffolds in the reference genome assembly, but because of the bias toward large scaffolds, they accounted for 23% of the genome sequence. The largest observed difference in MAC was 13, seen at 11 RAD sites (top 1%), all on different scaffolds (Fig 1). Of these, 10 had SNPs showing identical segregation patterns, with the minor allele absent in all 9 resistant lines, fixed in 6 of the 10 susceptible lines, and heterozygous in a 7th susceptible line. In other words, these 10 RAD sites showed mutually perfect LD (r = 1; p < 10^−5^) and seemed to comprise a large haplotype block, here designated the *RADres* genomic region (Table 3 and S1 Fig). The combined size of all ten scaffolds in the *RADres* region is 2.4Mb. The one remaining RAD site with a MAC difference of 13, on Scaffold7510, was in high but not perfect LD with *RADres* (r = 0.79, p < 10^−4^), with the minor allele homozygous in two additional lines, one resistant and one susceptible.

**Table 3:** Markers in the *RADres* region.

Name^a^	Type^b^	Scaffold^c^	Site^d^
*RADres1*	RAD tag SNP	LG24_random_Scaffold115	333090
*RADres2*	Indel in candidate gene	LG24_random_Scaffold332	87890
Unnamed	RAD tag SNP	LG24_random_Scaffold76	382528
Unnamed	RAD tag SNP	LGUN_random_Scaffold201	71351
Unnamed	RAD tag SNP	LG24_random_Scaffold332	292543
Unnamed	RAD tag SNP	LG24_random_Scaffold592	188090
Unnamed	RAD tag SNP	LG24_random_Scaffold788	80122
Unnamed	RAD tag SNP	LG24_random_Scaffold2400	13516
Unnamed	RAD tag SNP	LG24_random_Scaffold2452	30139
Unnamed	RAD tag SNP	LG24_random_Scaffold4819	41204
Unnamed	RAD tag SNP	LG24_random_Scaffold7794	3150

^a^Name of marker. Only named markers were assessed in outbred snails

^b^Type of polymorphism

^c^Scaffold in *B*. *glabrata* genome, BglaB1 assembly

^d^Site in *B*. *glabrata* genome, BglaB1 assembly

**Fig 2:**
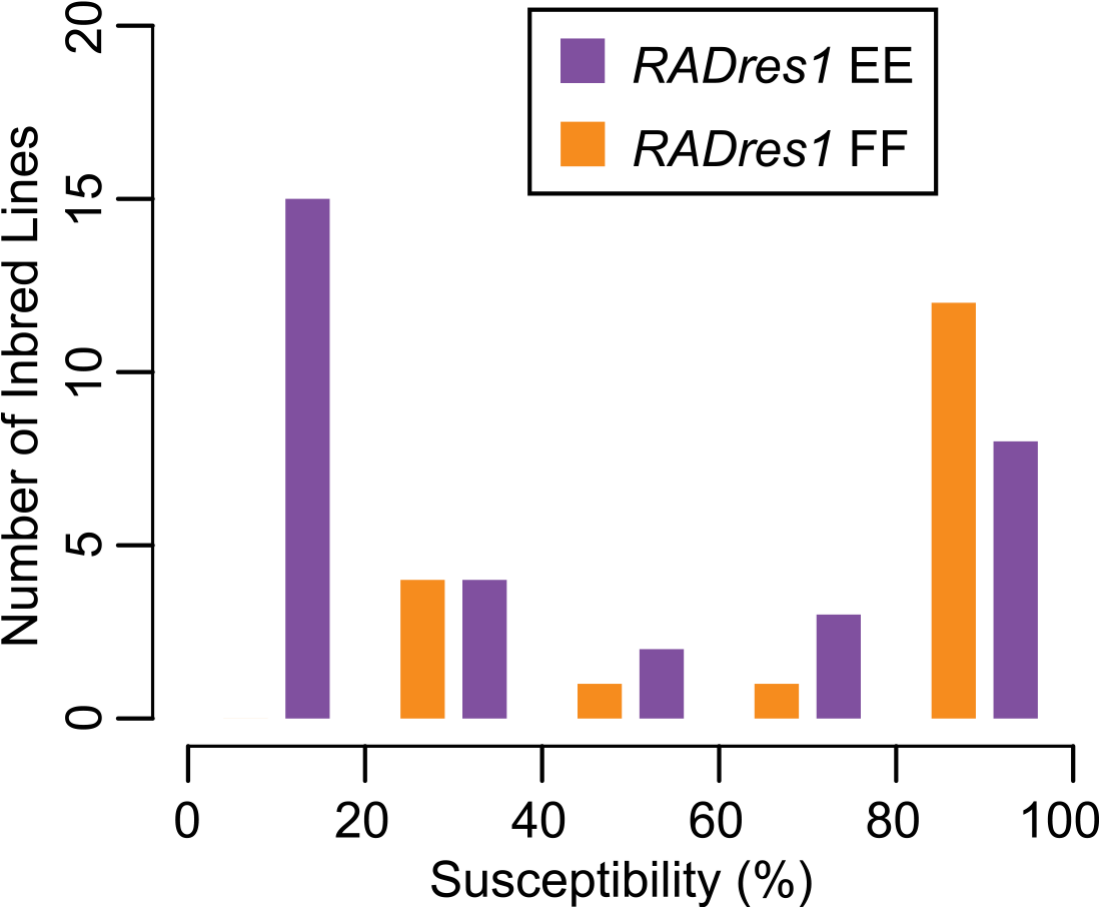
Influence of *RADres1* on susceptibility in 50 inbred lines. Lines are colored based on genotype (purple = EE, orange = FF) and binned based on susceptibility in windows of 20%. EE lines are more resistant on average. The most striking difference between genotypes occurs in highly resistant lines (susceptibility < 20%), which include no FF lines and 15 EE lines. One additional line (not depicted in figure) was heterozygous with 57% susceptibility.

We developed primers to amplify one of the *RADres* sites, on Scaffold115_333kb (“*RADres1*”, Tables 2 and 3), and we obtained genotypes from 51 inbred lines. We observed two alleles at *RADres1*, defined by a single C/T SNP at position 333090 (allele E = T; allele F = C; S2 Table). Allele E was associated with resistance and allele F with susceptibility, with mean susceptibility in EE lines (38%) half that of FF lines (76%) (t-test, p < 0.001; Fig 2). Among 19 resistant lines (<25% susceptibility), 18 were EE homozygotes and only one line with borderline susceptibility (22%) was an FF homozygote. All 15 highly resistant lines (< 20% susceptibility) were EE homozygotes. In contrast, among the 31 homozygous lines with > 25% susceptibility, 14 were EE and 17 were FF (Fisher’s exact test, p < 0.001). After excluding the 19 resistant lines, there was no relationship between genotype and phenotype among the 31 homozygous lines with > 25% susceptibility (mean EE susceptibility = 77%; mean FF susceptibility = 79%; t-test, p > 0.1).

In addition, we designed PCR primers to amplify a portion of another *RADres* scaffold, Scaffold332_88kb (“*RADres2*”), within an intron of a putative glycosyltransferase gene. This gene was of interest because glycosylation could be involved in self vs. non-self recognition. Therefore, we wanted to test whether *RADres2* showed a higher association with resistance than our original RAD marker, *RADres1*. We did not genotype the inbred lines at *RADres2*, but we used it in our assessment of outbred snails (see below).

### Characterization of *sod1* region in inbred lines

The *sod1* allele that correlates positively with resistance, allele B [24], had a MAC of 11 and a MAC difference of 5, so although it was more prevalent in resistant lines, as expected, it was not an outlier when compared to all RAD sites as was *RADres* (Fig 1). We observed 80 RAD SNPs at 46 RAD sites on 34 scaffolds showing perfect LD with the *sod1* B allele, including a RAD site at Scaffold10_584kb, very near the *sod1* position at Scaffold10_612-616kb. The total combined length of these scaffolds was 10.8Mb. Thus, *sod1* also resides in a large haplotype block, probably even larger than *RADres* (S2 Fig).

### Linkage disequilibrium analysis

**Fig 3:**
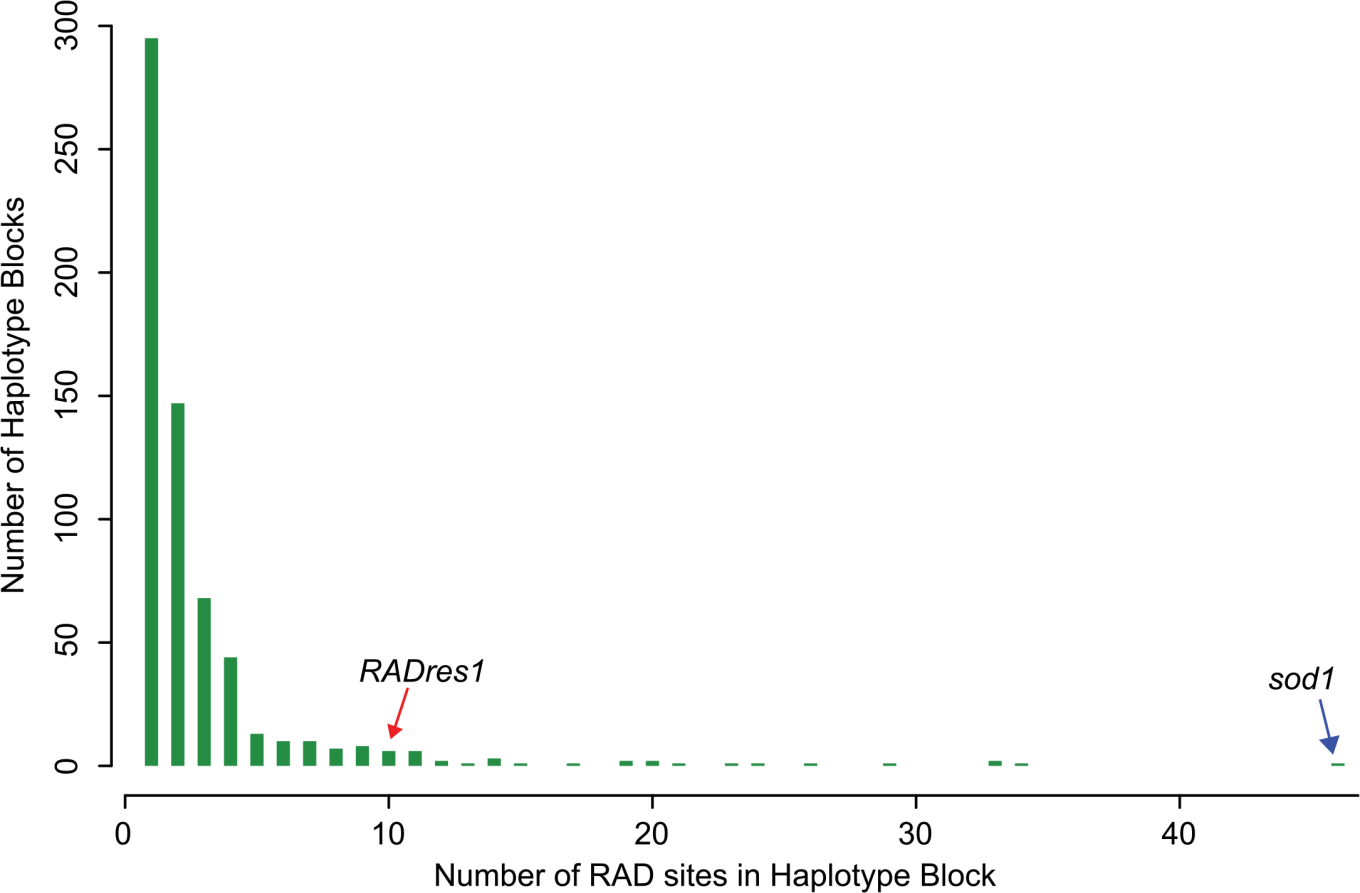
Extent of linkage disequilibrium (LD) in 13-16-R1. Among 19 inbred lines, RAD SNPs with identical genotype patterns (LD of 1) were grouped into haplotype blocks. The number of RAD sites represented by each haplotype block is shown. Most (82%) RAD sites show perfect LD with at least one other RAD site, indicating high genome-wide LD. The *RADres* block shows a typical extent of LD, with 10 RAD sites in perfect mutual LD. RAD sites with SNPs differentiating *sod1* B haplotype from the other *sod1* haplotypes form the single largest haplotype block in the genome (46 RAD sites). Thus, *sod1* appears to be in an unusually large haplotype block of perfect LD.

We observed relatively high genome-wide LD in 13-16-R1. Among the 340 RAD site pairs that were on the same scaffold and at least 0.1 Mb apart, 60% showed r > 0.75, and 42% showed perfect LD (r = 1) for at least one SNP. Among all RAD site pairs, including those on separate scaffolds, there were 7757 pairs showing perfect LD for at least one SNP, equivalent to each RAD site occurring in a cluster of 10 mutually identical RAD sites on average. There were 33 haplotype blocks that each contained at least 10 distinct RAD sites showing perfect mutual LD for at least one SNP, encompassing 32% of RAD sites (Fig 3). The single largest haplotype block was the *sod1* block described above with 46 RAD sites, and the second-largest block had 34 RAD sites. Thus, multi-scaffold haplotype blocks of high LD extending hundreds of kilobases or more are common in this laboratory population, and the extent of LD observed at *RADres* is not atypical, although the extent of LD observed at *sod1* is.

### Outbred snail genotypes

Of the 456 challenged outbred snails, we obtained phenotype and genotype data from 439 (S3 Table), with the remaining failures owing to snail death prior to phenotyping or insufficient extraction of DNA. In all 439 snails we obtained complete genotypes for six loci: *sod1*, *RADres1*, *RADres2*, *aif*, *infPhox*, and *prx1*. We also obtained genotypes for the majority of snails for two additional loci: *grc* (N = 278), and *bmplys* (N = 405). All loci were polymorphic with 2–4 alleles, and all had intermediate frequency alleles (25–75% frequency), thus conveying high power to detect both correlations with resistance and linkage disequilibrium among loci (Table 2). All loci were found to be in Hardy-Weinberg equilibrium (p > 0.05) except for *aif*, which showed a slight deficit of heterozygous genotypes (observe 216, expect 253; p < 0.0001), which could be explained by a rare null allele (~6% frequency) that by chance was never observed as a homozygote.

We observed significant LD between the two pairs of loci for which it was expected (S4 Table). The loci *sod1* and *bmplys* are separated by a physical distance of <500kb on the same scaffold. They are in high but imperfect LD, with the highest LD observed between the *sod1* B allele and *bmplys* (r = 0.91, p < 10^−15^). There were two *bmplys* alleles (J and K), with allele K at 37% frequency showing a positive correlation with the resistance allele B at *sod1*, which occurred at a similar frequency (33%). Almost all (99%) *sod1* B alleles co-occurred with *bmplys* K, and the remaining *bmplys* K alleles most commonly occurred with *sod1* A. Similarly, all three *RADres2* alleles showed significant high LD with *RADres1*, and allele G showed the highest LD with *RADres1* (r = 0.71, p < 10^−15^). Allele G at *RADres2* occurred at 71% frequency and was positively correlated with resistance allele E at *RADres1*, which occurred at a similar frequency (68%). The only other pairwise combination between alleles at different loci showing significant LD was between *sod1* D and *infPhox* S, which was marginally significant after Bonferroni correction (p = 0.04; S4 Table); because these two rare (≤ 2% frequency) alleles co-occur in only three individuals, and because the more common alleles at these loci do not show LD, we dismissed this result as unlikely to be biologically meaningful. No other pairwise combination between alleles at different loci showed significant LD (p > 0.05 for all).

### Outbred snail phenotype-genotype association

We observed 174 infected and 265 non-infected outbred snails (40% susceptibility). Our logistic regression analyses repeatedly showed the same main result: two genomic regions, (*sod1*/*bmplys*) and *RADres*, were significantly correlated with resistance, and no other loci were. We describe these results in detail below.

**Fig 4:**
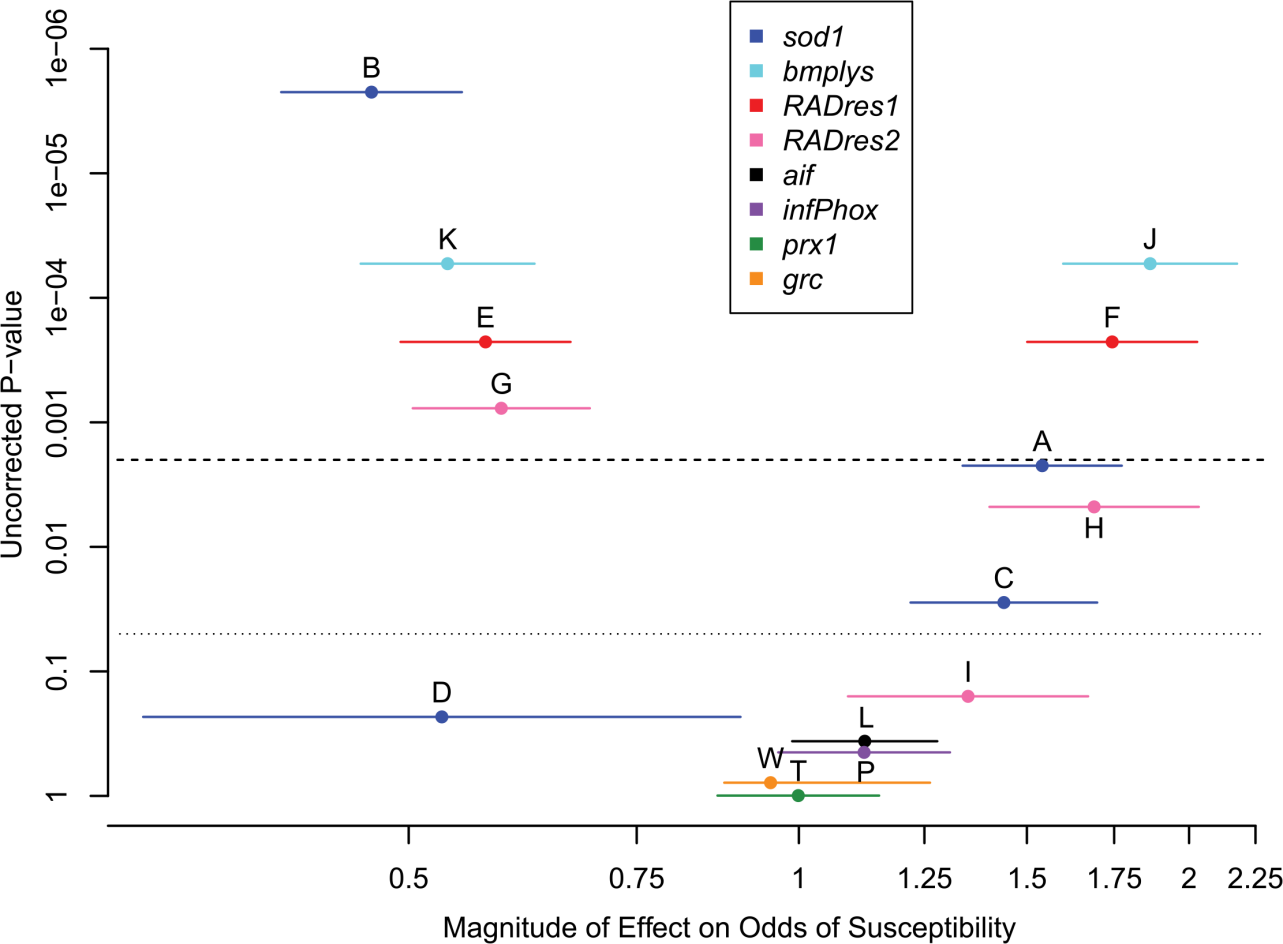
Effects of single alleles in outbred snails. We measured the effects of individual alleles using separate simple logistic regression analyses for each allele at all loci. Each allele is plotted according to its multiplicative effect on the odds of susceptibility (x-axis) and the p-value for its effect (not corrected for multiple tests; y-axis). Standard errors are shown with horizontal lines flanking each point. Alleles are colored based on locus. Only alleles at *RADres1*, *RADres2*, *sod1*, *and bmplys* are significant (uncorrected threshold of 0.05 indicated by dotted line, threshold after Bonferroni correction of 0.002 indicated by dashed line). Alleles are labeled (A-L, P, W, and T) as in Table 2. For non-significant loci, only the most common allele is shown.

**Fig 5:**
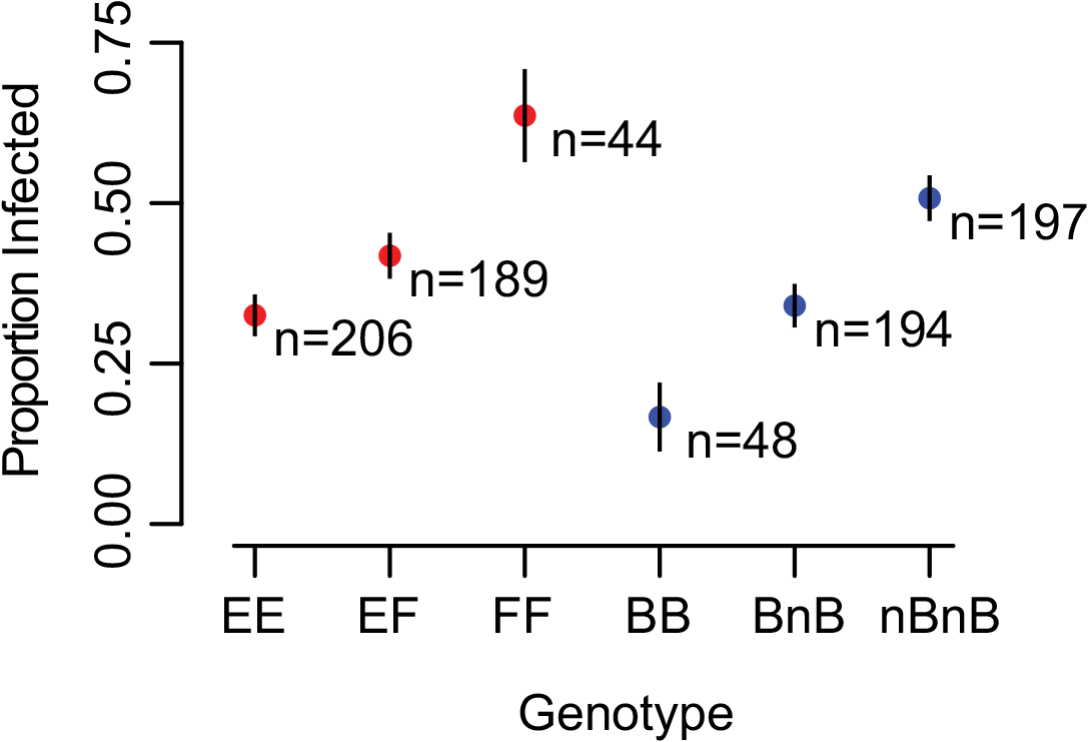
Independent influence of *RADres1* and *sod1* genotypes on resistance. Among 439 snails, both the E allele at *RADres1* and the B allele at *sod1* are positively correlated with resistance. For each locus (red genotypes = *RADres1*; blue genotypes = *sod1*), all three genotypes are significantly different from each other, indicating additive rather than dominant effects. Standard errors of proportions are indicated by vertical bars.

In the single-allele tests, at least one allele of *sod1*, *bmplys*, *RADres1*, and *RADres2* showed a significant correlation with resistance (p < 0.05 for each; Fig 4 and S5 Table). For all alleles correlated with resistance at these four loci, an additive model explained more of the phenotypic variation and had a lower Akaike information criterion (AIC) than the dominant model. Comparing within the two pairs of linked loci, *sod1* had a non-significantly stronger effect on resistance than *bmplys*, and *RADres1* had a non-significantly stronger effect on resistance than *RADres2* (Table 2 and Fig 4). No allele at any of the remaining loci (*aif*, *infPhox*, *prx1*, and *grc*) showed a significant association with resistance, whether modeled as dominant or additive (p > 0.1 for each; Fig 4). Thus, we found two independent genomic regions correlated with resistance in 13-16-R1, and in both regions the allele most strongly correlated with resistance (*sod1* B and *RADres1* E) acts additively, reducing the proportion of infected snails by approximately 16% with each additional copy (Fig 5).

**Fig 6:**
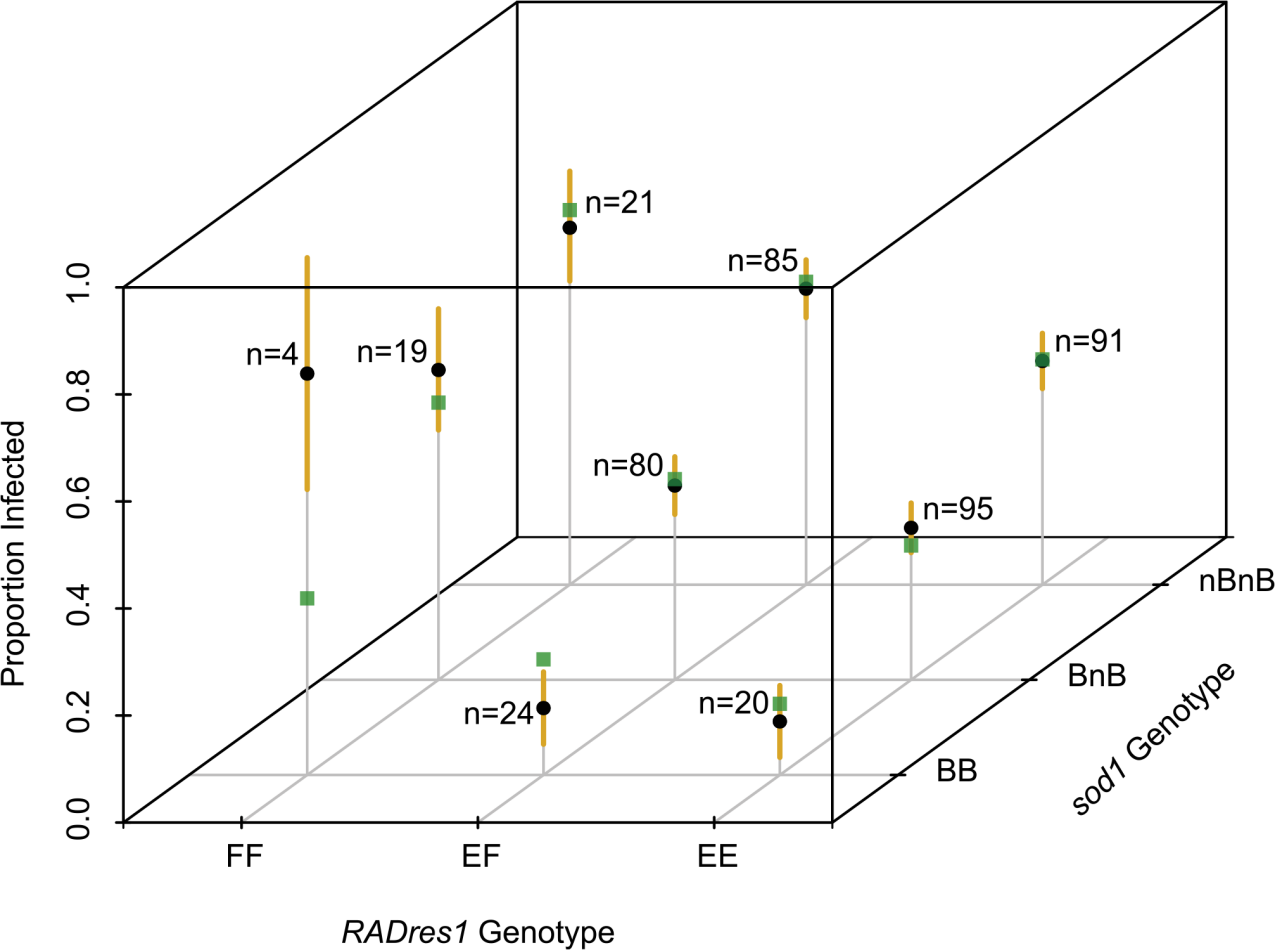
Joint influence of *RADres1* and *sod1* genotypes on resistance. Genotype combinations are indicated along the x and y axes. Empirical resistance values are plotted as black circles along the z axis. Standard errors of proportions for resistance at each genotype combination are shown with a vertical yellow line. Predicted values from an additive 2-locus multiple regression model with no dominance or epistasis are indicated with green squares. The data are consistent with this simple model, although minor non-additive effects may be responsible for small nonsignificant differences between predicted and empirical values.

Our results from the single-allele tests were mirrored in the two-allele tests, in that only alleles at *sod1*, *bmplys*, *RADres1*, and *RADres2* ever showed a significant correlation with resistance (p < 0.05) (Tables 2 and S6). For the two haplotype blocks with multiple loci and/or alleles (*RADres1* and the three alleles of *RADres2*; *bmplys* and the four alleles of *sod1*), we never observed a separate significant effect for a second allele after including the best allele for that block (*sod1* B or *RADres1* E). Although there is a trend that *sod1* D conveys resistance (Fig 4), it has no significant effect either alone or in a model that also includes *sod1* B. The single best fitting model included *sod1* B and *RADres1* E, which were also the two alleles with the strongest individual effects on resistance in their haplotype blocks (Fig 4). A simple additive model, in which alleles B and E contribute to resistance with no dominance and no epistasis, fits the data well (S6 Table and Fig 6), with both terms significant (p < 0.01 for both). This additive model explains 7% of the population variance in resistance, with each *sod1* B allele decreasing the odds of infection by 2.2 (95% CI: 1.58–2.99), and each *RADres1* E allele decreasing the odds of infection by 1.8 (95% CI: 1.32–2.43). Thus, the odds of infection for a snail with genotype BB/EE (predict 13% infected, odds = 0.15) are 16-fold lower than for a snail without a resistance allele at either locus (predict 70% infected, odds = 2.33). The genotype combinations with the poorest fit to this model (green squares in Fig 6) are BB/FF (predict 33% infected, observe 75% infected, N = 4) and BB/EF (predict 22% infected, observe 13% infected, N = 24). However, predicted values for all genotype combinations fall into the 95% Clopper-Pearson confidence intervals.

We tested for non-additive effects with more complex models, beginning with a test for dominance. If there were complete dominance, we would expect heterozygotes to show an identical impact on resistance as one of the homozygous genotypes. If there were partial dominance, the effects of heterozygotes might be intermediate between both homozygous genotypes, but more similar to one homozygous genotype than the other. For both *RADres1* and *sod1*, all three genotypes are significantly different from each other with respect to proportion of infected snails (Clopper-Pearson 95% confidence intervals do not encompass the proportions of other genotypes; Fig 5). Specifically, heterozygotes are intermediate in resistance; they are significantly more resistant than susceptibility allele homozygotes (FF or nBnB) and significantly less resistant than resistance allele homozygotes (EE or BB). Thus, for both loci we can reject the hypothesis of complete dominance. Observed heterozygous effects were not significantly different than the mean between the homozygous effects (encompassed in Clopper-Pearson 95% confidence intervals), indicating no evidence for partial dominance. Thus, we observe purely additive effects at both loci, rather than complete or partial dominance.

We then tested for epistatic interactions between loci. In a model with an additive effect of both *RADres1* and *sod1*, plus an interaction term, the interaction term was not significant (p > 0.1). To further explore the possibility of an interaction, we tested for an additive effect of each locus in the subset of individuals with each possible genotype at the other locus (Fig 6). The estimated reductions of odds of resistance of the *sod1* B allele in specified *RADres1* genetic backgrounds were as follows: FF background 1.0-fold (95% CI: 0.39–2.77), EF background 2.5-fold (95% CI: 1.58–4.10), EE background 2.2-fold (95% CI: 1.35–3.66), E- (EE or EF) background 2.4-fold (95% CI: 1.68–3.33). Thus, there is no significant effect of *sod1* in a FF background, but this is not significantly lower than the effect of *sod1* in an E- background (t-test, p > 0.1). The estimated reductions of odds of resistance of the *RADres1* E allele in a specified *sod1* genetic background were as follows: nBnB background 1.6-fold (95% CI: 1.06–2.53), BnB background 1.8-fold (95% CI: 1.12–2.78), BB background 4.4-fold (95% CI: 1.06–17.99), B- (BB or BnB) background 1.9-fold (95% CI: 1.23–2.92). Thus, *RADres1* has a significant effect in all *sod1* genetic backgrounds, with an effect in the BB background estimated to be higher, but not significantly so, than in the other genetic backgrounds. In summary, our data are consistent with an additive model (Fig 6), with no significant evidence for epistatic effects, although we may have low power to detect certain epistatic effects, especially those driven by BB/FF dual homozygotes of which we observed only four individuals.

## Discussion

We have identified a genomic region in *B*. *glabrata*, *RADres*, at which each copy of a resistance allele cuts the odds of schistosome infection in half. The impact of this locus is of similar magnitude to the only other known resistance locus in the 13-16-R1 snail strain, *sod1*, which also conveys an approximately 2-fold effect with each copy of a resistance allele. Thus, combined homozygosity at both loci confers a 16-fold change in the odds of infection (from approximately 2:1 odds to approximately 1:8 odds). Other candidate genes show no association with resistance, including the GRC [3] and three loci with constitutive expression patterns correlated with resistance in this population [23].

The *RADres* genomic region has not been previously implicated as relevant to snail immunity. Because the *RADres* region extends over several megabases, and likely includes many scaffolds that were not represented in our RAD-Seq data, the identities of candidate causal genes remain speculative. *RADres1* occurs on a scaffold that includes putative genes inferred to encode a methyltransferase, an ATP-dependent zinc metalloprotease, and an F-box/LRR-repeat protein. *RADres2* occurs in a putative glycosyltransferase gene, which could have an immune function due to the importance of glycoproteins in snail-trematode interactions [42]. The same scaffold also includes putative genes encoding an autophagy-related protein, a serine carboxypeptidase, and a single-pass transmembrane protein with no sequence similarity to other known proteins. One of the most studied immunity gene families in *B*. *glabrata*, the FREPs [43], does not occur among the known *RADres* scaffolds. Even without identifying the causal gene, we can make inferences about its probable function from the fact that *RADres* apparently acts additively. As a caveat, we acknowledge that the causal locus could act dominantly, with our observation of additivity being due to imperfect linkage disequilibrium between it and our markers (for example, *RADres1* EE homozygotes could be more resistant merely because they have two chances to share a haplotype with a rare dominant causal allele that occurs with only some E alleles). However, both our observation of high genome-wide LD and the admixed history of this laboratory population make this scenario unlikely. Thus, *RADres* seems to have a quantitative rather than qualitative effect, as resistance scales with the amount of its protein product, with homozygotes being more resistant than heterozygotes. *RADres* therefore represents a potentially rate-limiting step in the immune response, rather than a step with full functionality at low concentrations such as a recognition molecule that launches a signaling cascade after a single match to its target. If *RADres* encodes an immune effector like *sod1* does, the success of an immune response may depend on the quantity of this protein. Alternatively, the probability that an immune response is mounted at all may scale with the concentration of a signaling molecule produced by *RADres*, given the importance of signaling in snail defense [44]. In addition, two lines of evidence suggest that *RADres* contains a fundamental component of the immune system upon which other immune components depend. First, all inbred lines showing high resistance (< 20% susceptibility) are homozygous for the RADres resistance allele. Second, although there is no significant effect of *RADres1* genetic background on the effect of *sod1*, it is striking that the *sod1* B allele is not correlated with resistance among *RADres1* FF homozygotes (Fig 6). Because we observed only 4 BB/FF dual homozygotes, we have little power to test the role of *sod1* in this genetic background. However, the trend is that *RADres1* FF homozygotes are highly susceptible regardless of *sod1* genotype, suggesting that allele *RADres1* E may be required for *sod1* to function as an immunity gene. Thus, our data are consistent with the hypothesis that this population includes highly resistant genotypes (i.e. would nearly always resist infection with PR1 strain *S*. *mansoni* after indefinite trials) of which *RADres1* E is a necessary, though not sufficient, component.

The *sod1* locus was first identified as a candidate gene for *S*. *mansoni* resistance due to its crucial role in the oxidative burst [45]. It was subsequently shown that *sod1* genotypes correlate with resistance in 13-16-R1 [24] and that the resistance-associated B allele shows higher constitutive expression than the other alleles [46]. In this study we found a 2-fold effect of the B allele, the same magnitude reported by Goodall et al. [24]. Goodall et al. [24] also reported that the C allele is correlated with susceptibility, but they did not tease apart whether C is significantly different than A, or whether the link between C and susceptibility is simply the converse of the B/nB difference. Here, we did not detect an effect of C independent of B, suggesting that C and A are functionally equivalent. Intriguingly, our point estimate of infection odds reduction by *sod1* D, after accounting for *sod1* B and *RADres1* E, was quite high (3.5) but not significant (uncorrected p = 0.06). Our power to assess *sod1* D was limited as this allele was rare (2%) and never observed to be homozygous, but it is possible that this allele conveys a resistance impact even greater than *sod1* B or *RADres1* E. Because *sod1* was chosen as an *a priori* candidate, our working hypothesis is that this gene itself is the causal gene, not merely a linked marker for an unknown causal gene. However, there are several caveats to the putative causality of *sod1*. First, *sod1* resides on the largest high-LD haplotype block in this population, showing perfect LD with markers on many other scaffolds in the 19 inbred lines, suggesting that the region of association with resistance likely extends across many megabases encompassing numerous genes. Second, *sod1* is linked to at least two other genes (*cat* and *prx4*) that also contribute to the oxidative burst, both of which are in LD with *sod1* in the 13-16-R1 population [26]. These genes reside on contigs that were not represented by RAD-Seq sites, underscoring the fact that our RAD-Seq data likely underestimate the true size of the *sod1* haplotype block. Third, other potentially immune-relevant genes occur on contigs in this haplotype block, notably the *bmplys* gene encoding biomphalysin, a β pore-forming toxin which can directly kill *S*. *mansoni* sporocysts [38]. We have shown that *bmplys* is in high LD with *sod1* and both are therefore strongly correlated with resistance. If there is a genomic cluster of immunity genes, *sod1* is not necessarily the one harboring the functional polymorphism. On the other hand, the correlation with resistance is slightly, though not significantly, lower at *bmplys* than at *sod1*, suggesting that *sod1* may be closer to the causal center of this region of association. The fact that *sod1* was not an MAC difference outlier (Fig 1) underscores the limits of our statistical power with only 19 inbred lines and suggests that other loci of similar effect on host resistance may remain undetected. It is also possible that the effect of *sod1* is relatively weaker in highly inbred genetic backgrounds because non-additive genetic variance can play a larger role.

Although expression levels of *aif*, *infPhox*, and *prx1* are all positively correlated with resistance in this population [23], segregating polymorphisms at these loci show no phenotypic association. These three genes were initially targeted because of their potential immunological role. Specifically, *aif* encodes allograft inflammatory factor, associated with inflammatory response and activation of hemocytes [23]. Likewise, *infPhox*, encoding a putative phagocytic oxidase subunit of NADPH oxidase, and *prx1*, encoding peroxiredoxin, both likely affect the concentration of parasite-killing reactive oxygen species [23]. Our results do not invalidate the potential functional importance of the variation in expression at these loci, but show that it must be controlled in a trans, rather than cis, manner. That is, these genes are upregulated in response to a signal that ultimately depends on genetic variation elsewhere in the genome. For example, resistant individuals may have a transcription factor allele that binds more strongly to the motifs at *aif*, *infPhox*, and *prx1*. It is tempting to speculate that *RADres* in fact contains such a trans-acting variant. The strong additive effect of the E allele at *RADres1* could be due to its influence on expression of a large number of immune-relevant genes. Unfortunately, for the small number of inbred lines in which gene expression was assessed, we do not have the statistical power to dissect an association with *RADres* from the correlated association with resistance. Furthermore, the majority of phenotypic variation in resistance remains unexplained by the combination of *RADres* and *sod1*, so it is likely that other loci also play a role. A promising future direction will be to test for an effect of *RADres* genotype on genome-wide gene expression patterns.

The GRC genomic region is strongly correlated with *S*. *mansoni* resistance in *B*. *glabrata* from Guadeloupe [3]. Patterns of polymorphism at several genes in this genomic region in the Guadeloupean snails suggest long-term balancing selection, which would predict that functionally divergent alleles occur through the species range. However, we did not detect a correlation between GRC genotype and resistance in 13-16-R1. This negative result may be due in part to the different parasite strain used in this study (PR1) versus in Tennessen et al. [3] (Guadeloupian *S*. *mansoni*), since strain-by-strain (G x G) interactions are known to be highly variable in this host-parasite system [47–51]. In addition, GRC diversity may have been lost in the narrow laboratory bottleneck during the founding of 13-16-R1. That is, 13-16-R1 may have retained only functionally similar GRC alleles by chance, even if all founding populations harbored functional genetic diversity. Intriguingly, no locus in mixed-origin 13-16-R1 conveys as strong an effect on resistance as the GRC in the natural Guadeloupe population (8-fold impact on odds of infection, explains 23% of variation in trait, [3]). As snails likely coevolve with trematodes, it is plausible that many variants affecting immunity will become fixed as populations diverge, resulting in many resistance loci in mixed-origin populations like 13-16-R1, each of which determines a small fraction of phenotypic variation. In contrast, fewer loci will be segregating within a single population in the wild, and thus those that do harbor functional variants, like the GRC in Guadeloupe, will explain a large proportion of the phenotypic variation.

This research represents a step forward toward identifying resistance genes and immune mechanisms in snails that could be targeted in the control of global schistosomiasis. Although no one resistance allele is likely to be a “silver bullet” that could unilaterally block all schistosome infection [52], the host-parasite molecular interactions represented by these resistance loci may be exploited in combination with other strategies for disease control. We have identified in *B*. *glabrata* a new genomic region, *RADres*, with a strong influence on resistance to schistosomes. Genes in this region may be suitable for manipulation of immunity in wild snails, but further work is needed to fine-map the causal gene(s) and to determine their other possible phenotypic effects and their immune roles under different genetic backgrounds, parasite strains, or environmental conditions. We have also further characterized the genomic region harboring *sod1*, notably showing that linkage disequilibrium is particularly high in this region. Therefore, the causal gene could be any of the other linked genes nearby, not necessarily *sod1* itself, although because of its immunological role and expression patterns that vary by genotype [45],[46], *sod1* is still the best candidate in this genomic region. Finally, we have shown that several candidate genes known to be correlated with resistance either in other populations (GRC) or via expression patterns in this population (*aif*, *infPhox*, and *prx1*) do not contain genetic variants linked to resistance under the conditions of this study. These other loci could still be useful targets in manipulating the immunity of wild snails, but the *RADres* region may encode a candidate of similar or greater importance for generating the desirable phenotypic outcome of heightened resistance.

## Supporting Information

S1 Table Primers for loci assessed in this study. (XLS)

S2 Table Polymorphisms used to distinguish alleles. (XLS)

S3 Table Phenotypes and genotypes for 439 outbred snails. (XLS)

S4 Table Linkage disequilibrium among alleles in 439 outbred snails. (XLS)

S5 Table Results from single-allele tests. (XLS)

S6 Table Results from two-allele tests. (XLS)

S1 Fig *RADres* haplotype block. All SNPs on all scaffolds containing a *RADres* marker are included. Pairwise LD between SNPs is indicated by color; bright red indicates perfect LD. Sc = scaffold.
(PDF)

S2 Fig *sod1* haplotype block. All SNPs on all scaffolds showing perfect LD with the *sod1* B allele are included. Pairwise LD between SNPs is indicated by color; bright red indicates perfect LD. Sc = scaffold.
(PDF)
