# Supplementary figures and images for: Genome-Wide Scan and Test of Candidate Genes in the Snail Biomphalaria glabrata Reveal New Locus Influencing Resistance to Schistosoma mansoni
Source: PLoS Negl Trop Dis. 2015 Sep 15;9(9):e0004077. doi: 10.1371/journal.pntd.0004077 (PMC4570800; doi:10.1371/journal.pntd.0004077)

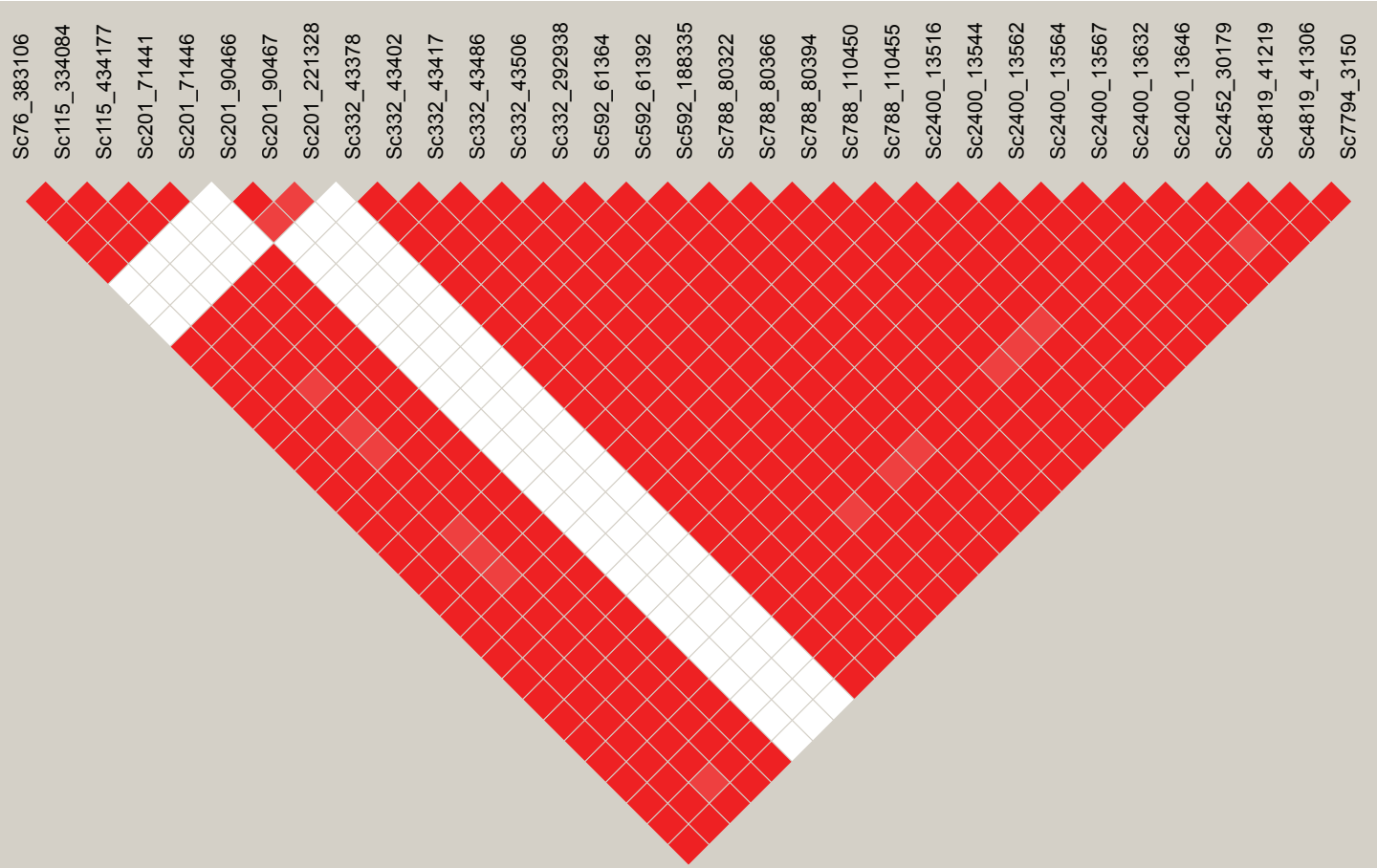

Supplement: S1 Fig — All SNPs on all scaffolds containing a RADres marker are included. Pairwise LD between SNPs is indicated by color; bright red indicates perfect LD. Sc = scaffold. (PDF) [file pntd.0004077.s007.pdf]

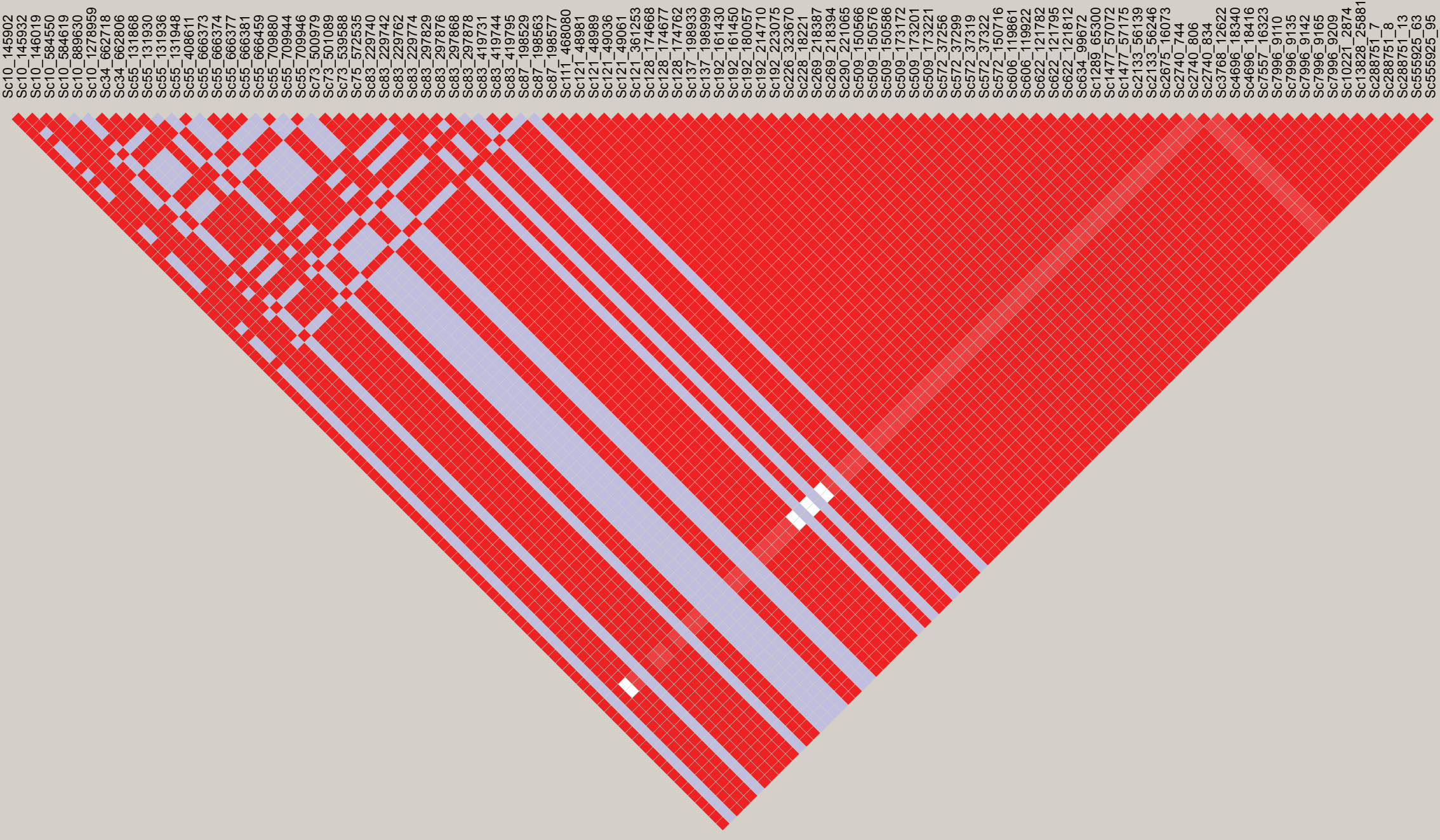

Supplement: S2 Fig — All SNPs on all scaffolds showing perfect LD with the sod1 B allele are included. Pairwise LD between SNPs is indicated by color; bright red indicates perfect LD. Sc = scaffold. (PDF) [file pntd.0004077.s008.pdf]
